# Supplementary material for: Heritability and genetic correlations of personality, life history and morphology in the grey mouse lemur (Microcebus murinus)
Source: R Soc Open Sci. 2019 Oct 30;6(10):190632. doi: 10.1098/rsos.190632 (PMC6837229; doi:10.1098/rsos.190632)
Supplement: Table S1 [file rsos190632supp1.docx]

**Supplementary Table 1**: summary of the covariance between morphological and early life traits**.**

|  | **Head width** | **Radius length** | **Birth weight** |
| --- | --- | --- | --- |
| **Radius length** | **Fixed effect: sex + age**  **COVa ± SE: 0.14 ± 0.067**  **P = 0.02**  **Ra = 0.73**  **COVr ± SE: 0.10±0.06** |  |  |
| **Birth weight** | Fixed effect: sex + age  COVa ± SE**:** 0.071 ± 0.064  P = 0.23  R = 0.46  COVr ± SE: 0.022±0.06 | Fixed effect: age  COVa ± SE**:** 0.067 ± 0.080  P = 0.16  Ra = 0.37  COVr ± SE: 0.028±0.068 |  |
| **Growth rate** | Fixed effect: age + sex  COVa ± SE**:** 0.032 ± 0.061  P = 0.58  Ra = 0.24  COVr ± SE: 0.14± 0.06 | **Fixed effect: sex + age**  **COVa ± SE: 0.15 ± 0.078**  **P < 0.01**  **Ra = 0.42**  **COVr ± SE: 0.17±0.07** | Fixed effect: sex  COVa ± SE**:** -0.09 ± 0.077  P = 0.22  Ra = -0.30  COVr ± SE: 0.12±0.07 |
