# Supplementary material for: Heritability and genetic correlations of personality, life history and morphology in the grey mouse lemur (Microcebus murinus)
Source: R Soc Open Sci. 2019 Oct 30;6(10):190632. doi: 10.1098/rsos.190632 (PMC6837229; doi:10.1098/rsos.190632)
Supplement: Table S2 [file rsos190632supp2.docx]

**Supplementary table 2:** microsattellite used for paternity assignments.

| Locus | Motif | Length (bp) | Annealing temp | Accession number | Reference | Primer F | Primer R | Alleles published for M. murinus |
| --- | --- | --- | --- | --- | --- | --- | --- | --- |
| Mm03 | (GA)18 | 93-149 | 55 | AF280081 | Radespiel et al. (2001) | AGCCTCACTGTTTCAGTTGTGT | GGCAGGAAATGTCATCTGG | 15 |
| Mm51 | (TG)10(TG)3 | 98–120 | 58 | AY154677 | Hapke et al. (2003) | CTTGAGGAAGTCTCTGAGG | TATCAAAATTGTAGCATGTAACA | 28 |
| Mm42 | (TG)25 | 123–197 | 58 | AY154675 | Hapke et al. (2003) | CATGGTTTCAGGTACTCCC | TCTTCATATCCATCTCTAATAC | 20 |
| Mm02 | (GA)18 | 142-172 | 53 | AF280080 | Radespiel et al. (2001) | TTAACAGGGCCTTCTCCTCAC | AATTGCCCAGTCCACACCT | 10 |
| Pvc 9.2 | (TG)17 | 143-167 | 46 | Not published | Wimmer et al. (2002) | ccc acc tat gct tag ttt g | tca ttt ccc aga gat aat c | 11 |
| Mm39 | (CT)6(AC)16(AT)5 | 153–221 | 58 | AY154673 | Hapke et al. (2003) | TACACTCTGGGTTACATAAGA | ATCTTTCATCTTCCTGTCCC | 13 |
| Mm30 | (CA)11 | 213–234 | 58 | AY154672 | Hapke et al. (2003) | GATGCTGAA CCT CTG TCT G | GGCATTTGCGCAAGGTCG | 26 |
| Mm21 | (A)17 | 213–245 | 58 | AY154669 | Hapke et al. (2003) | TCAATGCATCAATTAACCACG | CAGTTAACATCCTCAGCAATA | 16 |
| Efr 56 | (GTGC)2(GT)16 | 230-238 | 50 | Not published | Wimmer et al. (2002) | cca cct tag cat att tag cat | GTTTGATGTTCGGAACTGAGAG | 5 |
| Mm26b | (CA)19(CTCA)8 | 233–253 | 58 | AY154671 | Hapke et al. (2003) | TAAATAACCAAGTAAAGGGTTC | CTACAAATGGAATGGTGATGA | 15 |
